# The Effects of Acupuncture on Cerebral and Muscular Microcirculation: A Systematic Review of Near-Infrared Spectroscopy Studies

**DOI:** 10.1155/2015/839470

**Published:** 2015-06-11

**Authors:** Ming-Yu Lo, Ming Wei Ong, Wei-Yu Chen, Wei-Zen Sun, Jaung-Geng Lin

**Affiliations:** ^1^College of Chinese Medicine, China Medical University, No. 91, Xueshi Road, North District, Taichung City 404, Taiwan; ^2^Department of Anesthesiology, College of Medicine, National Taiwan University, 7 Chungshan South Road, Taipei 10002, Taiwan; ^3^National Research Institute of Chinese Medicine, Ministry of Health and Welfare, 155-1 Section 2, Linong Street, Beitou District, Taipei 11221, Taiwan

## Abstract

Acupuncture produces physiological effects via stimulating acupoints, proximal or distal to the region of effect. Near-infrared spectroscopy (NIRS) noninvasively measures tissue-level hemodynamics in real time. We review the literature investigating the effect of acupuncture on muscular and/or cerebral microcirculation. As the basis, we queried PubMed in June 2014 for articles mentioning both acupuncture and NIRS in title/abstract. The reviewed papers investigated either cerebral (*n* = 11) or muscular hemodynamics (*n* = 5) and, based on STRICTA for reporting acupuncture methodology, were overall poor in quality. Acupuncture was found to influence regional oxygen saturation in cerebral and muscular tissue. The cortical response in healthy subjects varied across studies. For subjects with stroke or cerebrovascular dementia, findings suggest that acupuncture may modulate dysfunction in cerebral autoregulation. The muscular response to pressure techniques was more intense than that to needling or laser. Probe proximity could impact measurement sensitivity. No one study simultaneously investigated the direct and remote responses. Research utilizing NIRS to investigate the hemodynamics of acupuncture presently lacks in scope and quality. Improved designs, for example, placebo-controlled, randomized trials, and standardized intervention reporting will raise study quality. Exploiting NIRS in clinical settings, such as stroke, migraine, or other pain conditions, is worthwhile.

## 1. Introduction

Acupuncture is the practice of stimulating specific points of the body (acupoints), most commonly by needling, with roots in traditional Chinese medicine, and aims to treat a wide range of ailments [1]. Physiological responses include analgesic and hemodynamic responses. The analgesic response, a reflection of the influence on the autonomic system, has been documented, although not without controversy [2–4]. The hemodynamic response is also of clinical interest. Reflexive responses include erythema, a local, relatively benign effect around the stimulation site, and syncope, a systemic, serious adverse effect in poorly compromised subjects [5–8]. These are rare but well known to acupuncturists. More commonly, the response is therapeutic and able to modulate autoregulation under pathological status, such as stroke and migraine [9–11].

Modes of acupuncture are several. Modern practice generally applies stainless steel needles. Variations are application of electricity to the needles, the use of laser at the acupuncture points, manual pressure at the points (acupressure), or moxibustion. Auricular acupuncture involves a collection of points/regions on the ear theorized in 1957 by Nogier [12–14]. Point locations may then be categorized by location (and tissue type): body (muscle/tendon), ear (cartilage), and scalp (subcutaneous tissue). Furthermore, the intended effects of acupuncture stimulation are generally proximal or distal. The distal effects depend on the meridian theory, while the proximal effects follow the theory of* A Shi* points, per Chinese traditional medicine [15–18].

The scientific mechanisms behind acupuncture have long been and still are mysterious in large part because the needling locations are often remote from the intended area of effect. Such responses to needling stimuli most likely arise from interactions within the nervous system, particularly the brain. Recent tools have made it easier to study these interactions in both muscular and cerebral tissues, from multiple angles. Near-infrared spectroscopy (NIRS) is one such tool that observes the hemodynamics at the tissue level. The muscular hemodynamics reflects the direct response, while the cerebral hemodynamics reflects the remote response to acupuncture stimulation.

NIRS observes tissue hemodynamics by using near-infrared light to monitor blood oxygenation in real time. It is a safe, noninvasive technique but has limited reading depth, while the breadth of the monitored region depends on the number and placement of probes [19–21]. Nonetheless, its portability, ease of use, and high temporal resolution are significant advantages over the more spatially comprehensive BOLD-fMRI (blood oxygenation level-dependent functional magnetic resonance imaging), while its ease of use and focus on microcirculation make it an attractive alternative to transcranial Doppler ultrasound (TCD), which focuses on blood flow, usually in specific arteries [10, 22–25].

These advantages lend themselves well to monitoring the immediate hemodynamic response in cerebral or muscular tissues to acupuncture stimulation. Our aim is to review the studies reporting the use of NIRS in investigating acupuncture, its effectiveness, and its mechanisms.

## 2. Materials and Methods 

**Figure 1:**
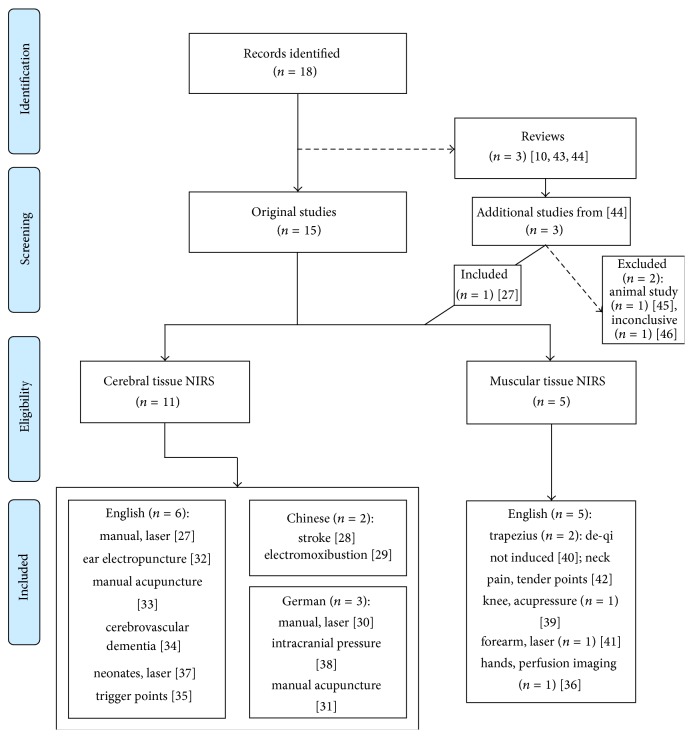
Flow diagram. Articles included for review targeted either cerebral hemodynamics or muscular hemodynamics with NIRS. No study attempted both.

We queried the PubMed database as of June 9, 2014, for all articles mentioning both acupuncture and NIRS in title or abstract, regardless of language. We included all original articles and excluded reviews. Any reviews were combed for relevant citations not found in the database search. For analysis, we focused on articles written in English or Chinese. Analysis of articles written in other languages was limited to abstracts and provided data and figures. All articles marked for analysis were obtained (Figure 1).

*Inclusion criteria are as follows:*

- being in PubMed database, up to June 9, 2014,
- mentioning “acupuncture” in title/abstract or as a MeSH term and “near-infrared spectroscopy (NIRS)” in title/abstract,
- being an original article,
- having no restriction on language,
- having, for analysis, language restricted to English or Chinese.

*Exclusion criteria are as follows:*

- it is a review;
- for analysis, languages other than English or Chinese were excluded, aside from abstract, tables, and figures.

*Methods of Analysis.* To assess study quality, we adapted the checklist for STRICTA (standards for reporting interventions in clinical trials of acupuncture) [26] (Table 1). Information on study designs, population, interventions, hemodynamic measures, and outcomes was organized in the tables (Tables 2 [Table tab3]–4). A summary table is also provided (Table 5).

## 3. Results and Discussion

Our query on June 9, 2014, produced (*n* = 18) results. We excluded (*n* = 3) reviews [10, 43, 44]. From a review, an additional three candidates, not covered in the database search, were added for consideration, of which only one was obtained and included [27, 44–46]. The two excluded are an animal study and a study involving two healthy subjects that observed changes in NIRS parameters (unspecified in the review) following acupuncture on ear, hand, and body [45, 46]. The articles ultimately included for review investigated either cerebral hemodynamics (CH) (*n* = 11) or muscular hemodynamics (MH) (*n* = 5) [27–42] (Figure 1).

### 3.1. Quality of Studies according to STRICTA

By STRICTA, the quality of studies under review may be considered poor in their reporting of acupuncture. We took a broad interpretation of acupuncture to include laser needling, moxibustion, and acupressure (Table 1). Details of needling, particularly number of needle insertions, depth of insertion, clarity between unilateral and bilateral application, and response sought to stimulation, were not reported in 44% of the studies (7 of 16) (Table 1, Item 2). Depth of insertion and response sought may not be applicable to some of these studies, since laser stimulation, electric stimulation, moxibustion, and acupressure were included, yet four (three) investigated manual needling among the seven underreporting depths of insertion (response sought) (Table 1, Items 2c and 2d). The number of needle insertions was often obscured from lack of distinction between unilateral and bilateral application. Regarding treatment regimen, the frequency of sessions, or time between sessions, was not reported in the majority of the studies (63%, 10 of 16) largely because most of these studies involved only one session (Table 1, Item 3). For other components of treatment, most of the studies did not have any additional interventions, as the subjects under investigation were generally healthy (Table 1, Item 4). Practitioner background for participating acupuncturists was fully reported in only 13% (2 of 16) of the studies. Three studies qualified participating acupuncturists as “expert” or “experienced” only. Of the remaining 11 studies, seven administered acupuncture but did not provide any description of the acupuncturists (Table 1, Item 5). Control or comparator interventions were also underreported (50%, 8 of 16), attributable to the majority (76%, 12 of 16) of these being observatory studies (Table 1, Item 6).

### 3.2. The Cerebral Hemodynamic Response

Five of 11 studies observed a significant increase in regional cerebral blood volume (rCBV) or oxyhemoglobin parameters. One involved a multisession, multipoint (body or body + scalp acupuncture) intervention for 20 stroke patients aged 41–75 and was one of two to record rCBV as the principal NIRS parameter [28]. The other investigated single-point electric moxibustion in 20 healthy subjects, aged 25–53, with mean 46 [29]. The principal oxyhemoglobin parameter in the remaining three studies was O_2_Hb measured by the NIRO 300 and involved healthy subjects aged 19–38. Two involved brief needle stimulations (20 s), with a retention time of 5 or 10 min [27, 30]. The other used continuous electrical stimulation on auricular acupuncture points, finding steady increase in O_2_Hb during each 15-minute stimulation of 100 Hz that persisted on level in the periods between stimulations [32].

It is likely that rCBV is synonymous with, or at least closely related to, total hemoglobin, as defined in Table 4. The interstudy populations assessed by rCBV were not comparable—one suffering stroke, the other, healthy—although both were older (age ranges: 41–75, 25–53) than the participants in the studies mentioned below [28, 29]. Among the studies finding increased oxygenation, one recorded the maximum amplitude of the changes in response to seven types of acupuncture stimulation (164 total) randomly distributed among 88 subjects [27]. The other two involved one or two subjects [30, 32]. All of these volunteers were healthy and aged 19–38. The stimulation times are comparable to the ones used in the studies finding oxygenation decreases. This complicates any attempt to draw a correlation between age and the cerebral hemodynamic response to acupuncture on healthy subjects.

Four of 11 detected significant decrease in oxyhemoglobin parameters. One involved a patient (age 77) with cerebrovascular dementia as a case study and found decreases after each NIRS-recorded session, coupled with increases in cerebral arterial mean blood flow velocity (measured by TCD). These decrements diminished in magnitude over the course of treatment (from a 13% decrease after the first treatment to a 4% decrease after the last) [34]. Another found oxygenation decrease in neonates after a single session of, but not during, laser acupuncture of Hegu LI 4. Peripheral oxygenation saturation (measured by means other than NIRS) was relatively constant, which implies that fractional tissue oxygen extraction increased [37] (see Table 4 for definition). The other two found decreases during brief stimulations (15 or 20 s) in subjects aged 19–30/19–45 (mean 23.5/23.9). The first of these correlated de-qi induction with the decreases in oxygenation in several areas of the brain, namely, the supplementary motor area, presupplementary motor area, and dorsomedial prefrontal cortex; the other observed decrease from manual needling at Hegu LI 4 but without clearly indicating induction of de-qi [31, 35].

Excepting the cerebrovascular dementia case, the populations are young and somewhat comparable in size (20, 20, and 16); also, the number of acupuncture points used is single to a few [31, 35, 37]. Manual needling showed quick response among healthy adults, but response to laser in neonates only emerged after the laser was turned off [31, 35, 37]. Also, needling stimulation was brief, comparable to the multiple-type acupuncture study finding increase in oxygenation, discussed above [27]. In spite of some common points among the studies investigating acupuncture in healthy young adults, the findings appear inconsistent: some found oxygenation increase; others found oxygenation decrease [27, 30–32, 35].

Two of 11 observed either a slight increase or no significant changes in oxyhemoglobin. Both of these also used transcranial Doppler ultrasound (TCD) to measure blood flow in the middle cerebral artery (MCA). The one finding no significant change in oxygenation generally found increased mean blood flow velocity in the left and right MCA (and, to a lesser degree, reduced pulsatility index) but no change in blood pressure parameters in response to (needle, pressure, or laser) stimulation of acupuncture points known to increase intracranial pressure in 34 healthy subjects, aged 20–35 (mean 25.2) [38]. The other study also found increased mean blood flow in the right MCA in response to an acupuncture scheme designed for “general increase of Qi-energy” in 12 subjects, aged 26–41 (mean 35.2) [33]. Aside from using both TCD and NIRS, too many parameters differ between the two to infer anything substantial.

In summary, the findings above indicate that the cerebral tissue oxygenation response to acupuncture, even in healthy young adults, varies widely, with no clear correlation to any single factor. Further research is required to investigate whether the variation in response carries over to subjects exhibiting dysfunction in cerebral autoregulation, as in stroke or migraine, since acupuncture has been found to have modulating effects [10, 47]. We recommend that future investigations consider the following for control: population age and fitness/health level; acupuncture type and intensity of stimulation (number of sessions, frequency, and duration); and NIRS machine model and recorded parameters and the number and positioning of probe(s).

### 3.3. The Muscular Hemodynamic Response

The response in the trapezius muscle was mixed between the two relevant studies. One found an increase in regional tissue oxygenation in the site of stimulation starting with needling, which stayed constant at least 5 min after stimulation ended, and identified no changes in a region centered 50 mm away [40]. The implication is that the direct oxygenation response to needling is detectable in the region surrounding the stimulation site, but not so in a region less than 1 cm away. The other found no increase but even a slight decrease in the ratio of oxyhemoglobin to total hemoglobin in the recorded region, which was located amidst six needles angled obliquely under the probe [41]. Some of the key differences between the two studies were population type (healthy versus “neck pain”), number of needles (1 versus 6), needling location (Jianjing GB 21 versus tender points), needling angle (vertical versus oblique), needle retention (2 versus 15 min), and NIRS parameters (oxyhemoglobin versus ratio of oxyhemoglobin to total hemoglobin).

A significant response in tissue oxygenation from acupressure stimulation of Xiyangguan GB33 was detected in the knee tissues on the stimulated side, while no significant response registered on the opposite side of the knee [39]. Acupressure may have a wider range of impact on muscular tissue oxygenation compared to manual or laser needling, simply owing to the nature of the techniques (pressure from the thumb versus needling at a point).

No significant response in tissue oxygenation was detected in the forearm from laser needle stimulation at Neiguan Pe 6, located 2 cm proximal to the middle point of the carpal fold between the tendons of M. flexor carpi radialis and M. palmaris longus [42]. The NIRS probe was located on M. flexor carpi ulnaris. Increased blood flow in a nearby region (5 cm proximal to the middle point of the carpal fold between the tendons of M. flexor carpi radialis and M. palmaris longus) was detected by laser Doppler spectroscopy. It is possible the NIRS probe was too distant from the site of stimulation or that laser needling may not have a strong enough effect for NIRS to detect a significant response.

A significant change in the perfusion rates of the hands was found in response to the first session of manual needling of Hegu LI 4 and Houxi SI 3. However, at the third session five days after, the responses failed to register significance. The baseline perfusion rates from the first to the third trial increased in one subject, but not the other [36]. This may reflect an acclimatization of muscular tissue perfusion to repeated acupuncture (three 10-minute sessions over 5 days). The study only has two subjects of different gender and age. No firm conclusions can be drawn from this study.

In summary of the MH studies, the technique of stimulation and proximity of the probe to the stimulation site appear to have a discernible impact on the detection and intensity of a response in the muscle and connective tissues. The findings suggest that in muscular tissue, acupressure has a greater impact on regional oxygenation than acupuncture, which in turn exceeds that of laser stimulation. Oxygenation has not been found to decrease in response to acupuncture, but the number of studies is few.

## 4. Conclusion 

Research using NIRS to investigate the hemodynamic effects of acupuncture is presently lacking in scope, number, and quality. Further studies may exploit the ease of use and real-time capacity of NIRS to monitor regional, tissue-level blood oxygenation to examine the concurrent response locally in the muscular tissue and remotely in the cerebral tissue. Improved study designs, accounting for the limitations of NIRS, placebo-controlled RCTs, and standardized reporting on interventions, such as adherence to STRICTA, will raise the quality of studies. Although the hemodynamic response to acupuncture varied widely among healthy subjects, it is worthwhile to extend the use of NIRS to clinical settings, such as stroke, neck pain, migraine, or other pain conditions.

## Figures and Tables

**Table 1:** Adherence to STRICTA. Articles organized by category according to a checklist provided by STRICTA.

Item	Detail	Provided		
		Yes	Unclear or incomplete	No or not applicable
(1) Acupuncture rationale	(1a) Style of acupuncture	16		
	(1b) Reasoning for treatment	11		5 [27–31]
	(1c) Extent to which treatment was varied	16		

(2) Details of needling	(2a) Number of needle insertions	9	7 [30–36]	
	(2b) Points used (uni/bilateral)	16; u/b: 9	u/b: 3 [29–31]	u/b: 4 [32–34, 36]
	(2c) Depth of insertion	9	1 [32]	6 [28–31, 34, 37]
	(2d) Response sought	9		7: [27, 29, 31, 32, 37–39]
	(2e) Needle stimulation	14		2 [33, 34]
	(2f) Needle retention time	14	2 [30, 31]	
	(2g-1) Needle (dimensions)	13		3 [29, 32, 39]
	(2g-2) Type (material/mfc)	12	3 [35, 36, 40]	1 [39]

(3) Treatment regimen	(3a) Number of sessions	13	3 [27, 30, 31]	
	(3b-1) Frequency or time between treatments	6 [27, 28, 34–36, 38]	2 [30, 32]	8
	(3b-2) Duration of treatment sessions	14	2 [30, 31]	

(4) Other components of treatment	(4a) Details of other interventions for the acupuncture group	2 [33, 34]		14
	(4b) Setting and context of treatment	16: N = 15 [27–35, 37–42]; T = 4 [32–34, 38]; P = 2 [33, 34]; O = 3: eeg [33], lds [42], icg [36]		

(5) Practitioner background	(5) Description of participating acupuncturists	2 [35, 36]	3 [32, 38, 39]	11

(6) Control or comparator interventions	(6a) Rationale for the control or comparator	2 [35, 42]	7 [27, 28, 30, 32, 33, 38, 40]	7 [29, 31, 34, 36, 37, 39, 41]
	(6b) Precise description of the control or comparator	8	2 [30, 33]	6 [31, 34, 36, 37, 39, 41]

(2b) u/b: uni/bilateral.

(2d) Acupressure [39], laser acupuncture [37], electric moxibustion [29], and P-Stim, a form of auricular electroacupuncture [32].

(2g) mfc: manufacturer; no mfc [35, 36, 40], material not mentioned [40].

(4b) All studies [27–35, 37–42], except [36] are conventional NIRS; N: NIRS, T: TCD, P: Pointselect (a tool to help identify acupuncture points), O: other; eeg: electroencephalogram, lds: laser Doppler spectroscopy, and icg: indocyanine green perfusion imaging (an application of NIRS).

**Table 2:** Study designs and populations. Summary of objectives, study design types, and populations involved in the reviewed articles.

Purpose	Study design	Population	Type (Pop.)
Compare body (A) versus body & scalp (B) acupuncture for stroke [28]	Comparative	*n* = 20: A/B = 10/10, f : m = 4 : 6/3 : 7, by intervention, age range 41–72/42–75	Stroke

Electric moxibustion at (a) Baihui GV 20 or (b) Shenque CV 8 for healthy subjects [29]	Comparative	*n* = 20 (a) *n* = 10, f/m = 5/5, age range 25–53; (b) *n* = 10, f/m = 5/5, age range 27–51	Healthy

Changes in regional cerebral oxygenation after various methods of acupuncture [27]	Observational	*n* = 88: f/m = 50/38, age range 19–38	Healthy

Effects of manual and laser acupuncture on cerebral oxygenation [30]	Observational	*n* = 3, male, ages 25, 50, and 70	Healthy

P-STIM auricular electroacupuncture [32]	Observational	*n* = 2, female, ages 23 and 27	Healthy

Regional cerebral oxygenation changes during and after acupuncture [33]	Observational	*n* = 12: f/m = 4/8, age range 26–41	Healthy

Cerebral parameters of healthy subjects after stimulating acupuncture points associated with intracranial pressure [38]	Observational	*n* = 34, f/m = 24/10, age range 20–35 Intensive care patient after severe head injury (*n* = 1, age 15)	Healthy

Acupuncture for cerebrovascular dementia [34]	Case study	*n* = 1, age 77, female	Cerebrovascular dementia

Changes in regional cerebral oxygen saturation in neonates undergoing laser acupuncture at Hegu LI 4 [37]	Observational	*n* = 20: f/m = 8/12, age < 1	Neonates

Effects on brain activity of trigger point (TP) versus nontrigger point stimulation and de-qi induction [35]	Comparative	*n* = 20: f/m = 5/15, age range: 19–30 TP first: f/m = 1/9, non-TP first: f/m = 3/7	Healthy

Effects of acupuncture at Hegu LI 4 on central frontal cortex [31]	Observational	*n* = 16, f/m = 9/7, age range 19–45	Healthy

Compare blood oxygenation in stimulation region and distant region in trapezius muscle [40]	Controlled	*n* = 19: AS *n* = 9: f/m = 7/2 age 36 no AS *n* = 10: f/m = 7/3, age 29	Healthy, acupuncture-experienced

Tender dry point needling for neck pain (katakori) Experiment I [41]	Controlled	*n* = 9: f/m = 7/2, age range 22–48; control *n* = 4: f/m = 0/4, age range 25–27	Neck pain

Tender dry point needling for neck pain (katakori) Experiment II [41]	Observational	*n* = 13: f/m = 8/5, age range 24–48	Neck pain

Effect of acupressure at Xiyangguan GB 33 on regional oxygen saturation of deeper knee tissues [39]	Observational	*n* = 12: f/m = 5/7, age: 23.8 ± 1.6 yrs	Healthy

Effect of laser needle stimulation at acupuncture point on blood flow and oxygenation in forearm [42]	Randomized Double-blinded Placebo-controlled	*n* = 33: age 26.6 (3.4) laser/no-laser = 18/15	Healthy

Near-infrared optical imaging to evaluate efficacy of acupuncture on peripheral tissue perfusion [36]	Observational	*n* = 2: f/m = 1/1, age 20/39	Healthy

**Table 3 tab3:** Acupuncture interventions. Summary of the acupuncture interventions, including placebo treatments. No medications, except in one case study, were involved in these studies [34].

Technique	Duration	Session (s)	Retention time	Points	De-qi
MB [28]	37 days	22-23 sessions/37 days: 15/15 + 0/7 + 7-8/15	30 min: every 5 min, apply 1 min stim (6x)	Varies with symptom and timing of treatment (3–15 points)	Yes
MB + S [28]	37 days	15/15 + 0/7 + 7-8/15 sessions/days	30 min: every 5 min, apply 1 min stim (6x)	Varies with symptom and timing of treatment (7–22 points)	Yes
EX [29]	1 day	1	30–40 min: 2 × 15 min stim + 5 min rest btw	One of GV 20 or CV 8	
MB, MA, MH, C, C + L, C + L′, Pt^*^ [27]	1 day	1.86 (avg.), >30 min btw, randomized^*^	10 s stim + 10 min (retention or laser)	MB: BL 2, Ex-HN 4; MA: “eye” (ear) and “liver” (ear); MH: Yandian, “eye” (E2) Korean hand points; C: all the above; Pt: placebo point^*^	
Light stimulation, MB, L, Pt [30]	1 day	4: one of each	20 s stim of each	LI 4, St 36, BL 60, BL 65, BL 66, BL 67	Yes
L, Pl [30]	1 day	2: one of each	20 s stim of each	GB 14, PC 6	
L, Pl [30]	1 day	2: one of each	20 s stim of each	GB 14, PC 6, CV 6, St 36, SP 6, LV 3	
EA [32]	1 week	4 sessions of different stim patterns	Several hours: varied (5, 15 min, or 3 hr stim)	Ear points: “eye” and “liver”	x
MB [33]	1 day	1	20 min retention after de-qi	PC 6, CV 6, ST 36, SP 6	Yes
MB, R, L [38]	1 day	3: randomized, one of each, >10 min btw	5 min for each (MB: 20 s stim + 2 min btw)	St 7, SJ 22	
MB + L [34]	13 weeks	11 (10 needle + 1 laser) sessions/13 weeks	20 min	He 5, He 7, Sp 6, BL 10, BL 17, BL 23, St 36	Yes
L [37]	1 day	1	5 min stim + 10 min undisturbed	LI 4	
MB [35]	1 day	2: 5 min btw alternate types	13 min: 3 min after insert, 8 × (15 s stim + 1 min no stim)	2 types^**^: TPs with de-qi; non-TPs with or without de-qi	Yes
MB [31]	1 day	1	6 min: 2 × 20 s stim + 5 min btw	LI 4	
MB [40]	1 day	1	2 min	GB 21	No
MB [41]	1 day	1	15 min	Tender points of the trapezius (6 needles obliquely inserted)	
MB [41]	2 days	1	15 min	Tender points of the trapezius (6–10 needles perpendicularly inserted)	
R [39]	1 day	1	5 min	GB33	
L [42]	1 day	1	10 min	Pe 6	
Pl [42]	1 day	1	10 min	Pe 6	
MB [36]	7 days	3	10 min	LI 4, SI 3	Yes

C: combination acupuncture; EA: electroauricular acupuncture; EX: electromoxibustion; L: laser acupuncture; L′: laser at 30% greater intensity; MA: manual auricular acupuncture; MB: manual body acupuncture; MH: manual hand acupuncture; Pl: placebo laser (laser off); Pt: placebo point needling; R: manual acupressure; S: scalp acupuncture; btw: between; stim: stimulation; min: minutes; s: seconds; 164 total sessions of 7 possible types of acupuncture randomized among the recipients (*n* = 88); the number of instances of each type (MB, MA, MH, C, C + L, C + L′, and Pt) is 23, 23, 23, 27, 27, 18, and 23, respectively. The placebo point was located 6 cun above the wrist on the radial ledge, off the lung meridian in the forearm.

^**^Trigger points (TPs) are located in the right extensor muscle of the forearm; non-TPs are 2 cm away from TPs. De-qi was induced from all TPs, but not all non-TP stim's.

**Table 4:** NIRS results. Summary of hemodynamic outcomes as measured by NIRS.

NIRS	Measure	Anatomy	Time frame	Outcomes^†^
Not available [28]	rCBV	Prefrontal cortex	At the (A) 0th, (B) 10th, (C) 20th, (D) 30th min of 30-minute acupuncture	During MB, rCBV ↑ at growing rate (130% ↑ from A to D); during MB + S, rCBV ↓ at B, then ↑ at C and D (136% ↑ A to D). At A, base MB + S >base MB
Not available [29]	rCBV	Prefrontal cortex	At the 0th, 10th, 20th, 30th min of EM^†^	rCBV ↑ during intervention
NIRO 300 [27]	O_2_Hb, HHb^††^	Prefrontal cortex	During 10-minute needle retention/laser and a period 5 min after stim	O_2_Hb ↑ and HHb ↓ during MH, MB, C, C + L′; O_2_Hb ↑ and HHb ↓ slightly from Pt; O_2_Hb ↓ and HHb ↑ slightly from A. Same response at least 5 min after
NIRO 300 [30]	O_2_Hb, HHb, t-Hb, CtOx, TOI	Central cortex (crown of head)	During all stim and rest periods between (20 s) stim's	O_2_Hb ↑ and TOI ↑ from MB or L. Response to MB >Response to L. O_2_Hb • and TOI • from needling or laser of Pt
NIRO 300 [32]	O_2_Hb	Frontal areas of brain	Before and during all stimulation periods	O_2_Hb ↑ each time during 15-minute EA stim of 100 Hz on “eye” acupuncture points
INVOS 3100 [33]	rSO_2_ (NIRS)	Forehead	(A) 10 min before, (B) 2 min into, (C) 10 min after (20 min) needling	rSO_2_↑ slightly at B and C
INVOS 5100 [38]	rcSO_2_	Prefrontal cortex	1 min before, 3 min into, and 1 min after	rcSO_2_•
INVOS 5100 [34]	rcSO_2_	Prefrontal cortex	Before and 10 min after needling, for the 1st, 2nd, 3rd, and 11th sessions	rcSO_2_↓ (4%–13%) after each needling. Magnitude of change at each session reduced with successive sessions
NIRO 300 [37]	rcSO_2_ SpO_2_ cFTOE	Prefrontal cortex	3 × 5-minute sampling periods: before, during, and after laser stim	rcSO_2_↓, SpO_2_•, and cFTOE ↑ in the postintervention period. No changes before or during stimulation
fNIRS: 2 × OMM 3000 [35]	O_2_Hb	Whole cortex	11 min: 2 min after needle insertion to end of acupuncture	Over 20 s interval, in SMA, pre-SMA, and mPFC: O_2_Hb ↓ during and 5 s after de-qi stim's; O_2_Hb • during stim with no de-qi. O_2_Hb • in the other cortical regions
NIRO 300 [31]	O_2_Hb, HHb, CtOx	Central region of cortex	7 min: 1 min before to end of acupuncture	After each stim, O_2_Hb ↓ HHb ↑ CtOx •

HEO-200 [40]	O_2_Hb, HHb, t-Hb	Trapezius muscle^**^	From 3 min before to 5 min after non-de-qi stim	O_2_Hb ↑ t-Hb ↑ HHb • in stim region during and after stim compared with distant region. Parameters for controls •
OM-200 [41]	t-Hb SdO_2_	Trapezius muscle	5 min before to 5 min after needling	t-Hb • SdO_2_• after needling
OM-200 [41]	*T*_*R*_	Trapezius muscle	Before, during, and after 1-minute exercise	*T*_*R*_ ↓ one day after needling in 10/13 patients
INVOS 5100 [39]	rSO_2_	Knee	Just before, 2 min into, and immediately after	rSO_2_↑ during and after stim on the stim side (*P* = 0.033); rSO_2_• on opposite side
InSpectra [42]	O_2_Hb and t-Hb	Forearm	4 × 2-minute sampling periods over 14 min: before, during, and after	O_2_Hb • t-Hb • in the sampling periods
NIR imaging: Vas View [36]	Perfusion rate	Hands	4 × 15 min: 10 min before (A_1_, A_3_) and after (B_1_, B_3_) 1st and 3rd MB^†^	B_1_: perfusion ↑. A_1_ versus A_3_ baselines: one case ↑, but the other •. B_3_: perfusion •

↑: significant increase; ↓: significant decrease; •: insignificant or no change; stim: stimulation; rCBV: regional cerebral blood volume; rSO_2_: regional oxygen saturation; rcSO_2_: regional cerebral oxygen saturation; cFTOE: cerebral fractional tissue oxygen extraction, calculated by (SpO_2_ − rcSO_2_)/SpO_2_, where SpO_2_ is peripheral oxygen saturation; O_2_Hb: concentration of oxyhemoglobin; HHb: concentration of deoxyhemoglobin; t-Hb: total hemoglobin (Δt-Hb = ΔO_2_Hb + ΔHHb); CtOx: concentration of cytochrome oxidase aa3; TOI: tissue oxygenation index; SdO_2_: oxygenation rate (%) calculated by ΔO_2_Hb/Δt-Hb; *T*_*R*_: half recovery time of SdO_2_ after maximum exertion of trapezius for 1** **min; SMA: supplementary motor area; mPFC: dorsomedial prefrontal cortex.

INVOS 3100, 5100: Somanetics, Troy, USA; NIRO 300: Hamamatsu, Japan; OM-200 (number P/N 101-40200), OMM 3000: Shimadzu Co. Ltd, Kyoto, Japan; Model HEO-200: OMRON Ltd. Inc., Japan; Vas View: Vieworks Corp., Seongnam, Gyeonggi-do, South Korea; InSpectra: Hutchinson Technology Inc., Netherlands.

^†^Abbreviations for interventions are in Table 3. ^††^Maximum amplitude of the changes in oxyhemoglobin and deoxyhemoglobin.

^*^On midpoint between the C7 spinous process, near neck tender points; ^**^near and 50 mm away from Jianjing GB 21 stimulation point.

**Table:** (a)

Population			Stimulation type	Parameter^†^	Response
Type	Size (f : m ratio)	Age (mean)			
Stroke [28]	20 (7 : 13)	41–75	Needling: multipoint, multisession, intensive	rCBV	+
Healthy [29]	20 (10 : 10)	25–53 (46)	Electric moxibustion: single point, single session	rCBV	+
Healthy [27]	88 (50 : 38)	19–38 (25.7)	Needling or needling + strong laser: multipoint, 1.86 average sessions/subject^a^	O_2_Hb	+
Healthy [30]	1 m	25	Needling: multipoint	O_2_Hb	+
Healthy [32]	2 f	23, 27	Electrical ear stimulation^b^	O_2_Hb	+
Healthy [33]	12 (4 : 8)	26–41 (35.2)	Needling: single session, multipoint	rSO_2_ (INVOS 3100)	0+
Healthy [38]	34 (24 : 10)	20–35 (25.2)	Separate needling, acupressure, laser at two points (ICP)	rcSO_2_ (INVOS 5100)	0
Dementia^c^ [34]	1 f	77	Needling: multisession, multipoint	rcSO_2_ (INVOS 5100)	−
Neonates [37]	20 (8 : 12)	<1	Laser: single session, single point	O_2_Hb	−
Healthy [35]	20 (5 : 15)	19–30 (23.5)	Needling: trigger points and nontrigger	O_2_Hb (OMM 3000)	−/0^d^
Healthy [31]	16 (9 : 7)	19–45 (23.9)	Needling: single session, single point	O_2_Hb	−

+: significant increase; 0: no significant change; −: significant decrease; 0+: slight increase in parameter.

^†^The parameter measures either tissue-level oxygenation or regional blood volume. See Table 4 for definitions. Except for one case, all measurements for O_2_Hb use the NIRO 300.

^a^164 instances of acupuncture chosen from 7 possible schemes (including placebo needling) randomly applied to the pool of 88 subjects.

^b^Electrical ear stimulation at a frequency of 100 Hz.

^c^Cerebrovascular dementia.

^d^Oxygenation response significant only during de-qi-inducing stimulations.

**Table:** (b)

Population			Target	Stimulation	Probe location	Parameter	Response
Type	Size (f : m)	Age (mean)					
Healthy [40]	9 (7 : 2)	(36)	Trapezius	Needling: single point	Needling at center of probe	O_2_Hb HHb t-Hb	+ + 0
Neck pain [41]	9 (7 : 2)	22–48 (35.1)	Trapezius	Tender point dry needling	Needles angled under probe^a^	t-Hb SdO_2_	0
Neck pain [41]	13 (8 : 5)	24–48 (36.5)	Trapezius	Tender point dry needling	During exercise^b^	*T*_*R*_	−
Healthy [39]	12 (5 : 7)	(23.8)	Knee	Acupressure: single session, single point	Near stim point and away^c^	rSO_2_	+
Healthy [42]	33 m	(26.6)	Forearm	Laser: single session, single point	M. flexor carpi ulnaris^d^	O_2_Hb t-Hb	0 0
Healthy [36]	2 (1 : 1)	20, 39	Hand	Needling: 3 sessions, two points	Whole hand	Perfusion rate	0+

+: significant increase; 0: no significant change; −: significant decrease; 0+: slight increase in parameter.

^a^Six needles angled obliquely to 20 mm under the center of the probe.

^b^ *T*_*R*_ is calculated during maximal exertion of trapezius conducted once before and again one day after needling. Needles angled perpendicularly. See Table 4 for definition.

^c^Two probes: one 2 cm from the stimulation point at Xiyangguan (GB 33) and the other on the opposite side of the patella.

^d^The stimulation site, Neiguan (Pe 6), is located 2 cm proximal to the midpoint of the carpal fold between the tendons of M. flexor carpi radialis and M. palmaris longus.

## References

[1] Liu G., Ma H. J., Hu P. P. (2013). Effects of painful stimulation and acupuncture on attention networks in healthy subjects. *Behavioral and Brain Functions*.

[2] Lee M. S., Ernst E. (2011). Acupuncture for pain: an overview of cochrane reviews. *Chinese Journal of Integrative Medicine*.

[3] Chang S. (2013). The meridian system and mechanism of acupuncture—a comparative review. Part 2. Mechanism of acupuncture analgesia. *Taiwanese Journal of Obstetrics and Gynecology*.

[4] Ernst E., Lee M. S., Choi T. Y. (2011). Acupuncture: does it alleviate pain and are there serious risks? A review of reviews. *Pain*.

[5] He W., Zhao X., Li Y., Xi Q., Guo Y. (2012). Adverse events following acupuncture: a systematic review of the chinese literature for the years 1956–2010. *Journal of Alternative and Complementary Medicine*.

[6] MacPherson H., Thomas K., Walters S., Fitter M. (2001). A prospective of adverse events and treatment reactions following 34,000 consultations with professional acupuncturist. *Acupuncture in Medicine*.

[7] Ernst E., White A. R. (2001). Prospective studies of the safety of acupuncture: a systematic review. *The American Journal of Medicine*.

[8] Zhu H. (2014). Acupoints initiate the healing process. *Medical Acupuncture*.

[9] Zhang J.-H., Wang D., Liu M. (2014). Overview of systematic reviews and meta-analyses of acupuncture for stroke. *Neuroepidemiology*.

[10] Lo M.-Y., Lin J.-G., Ong M. W., Sun W.-Z. (2013). Cerebral hemodynamic responses to acupuncture in migraine patients: a systematic review. *Journal of Traditional and Complementary Medicine*.

[11] Linde K., Allais G., Brinkhaus B., Manheimer E., Vickers A., White A. R. (2009). Acupuncture for migraine prophylaxis. *Cochrane Database of Systematic Reviews*.

[12] Gori L., Firenzuoli F. (2007). Ear acupuncture in European traditional medicine. *Evidence-Based Complementary and Alternative Medicine*.

[13] Nogier P. (1972). *Treatise of Auriculotherapy*.

[14] Nogier P. (1956). Le pavillon de l'oreille. Zones et points réflexes. *Bulletin de la Société d'Acupuncture*.

[15] Chang S. (2012). The meridian system and mechanism of acupuncture—a comparative review. Part 1: the meridian system. *Taiwanese Journal of Obstetrics & Gynecology*.

[16] Longhurst J. C. (2010). Defining meridians: a modern basis of understanding. *Journal of Acupuncture and Meridian Studies*.

[17] Lu D. H. (2000). *Muscle Injuries and Pains Involving Back and Limbs: Clinical and Experimental Studies on Acupuncture Treatment of Muscle Injuries*.

[18] Zhang S.-J. (2013). Origin and development of Ashi point locating method. *Chinese Acupuncture & Moxibustion*.

[19] Owen-Reece H., Smith M., Elwell C. E., Goldstone J. C. (1999). Near infrared spectroscopy. *British Journal of Anaesthesia*.

[20] Soul J. S., du Plessis A. J. (1999). New technologies in pediatric neurology. Near-infrared spectroscopy. *Seminars in Pediatric Neurology*.

[21] Murkin J. M., Arango M. (2009). Near-infrared spectroscopy as an index of brain and tissue oxygenation. *British Journal of Anaesthesia*.

[22] Fu T. C., Wang C. H., Lin P. S. (2013). Aerobic interval training improves oxygen uptake efficiency by enhancing cerebral and muscular hemodynamics in patients with heart failure. *International Journal of Cardiology*.

[23] Ogawa S., Lee T. M., Kay A. R., Tank D. W. (1990). Brain magnetic resonance imaging with contrast dependent on blood oxygenation. *Proceedings of the National Academy of Sciences of the United States of America*.

[24] Ogawa S., Menon R. S., Tank D. W. (1993). Functional brain mapping by blood oxygenation level-dependent contrast magnetic resonance imaging. A comparison of signal characteristics with a biophysical model. *Biophysical Journal*.

[25] Markus H. S. (2000). Transcranial Doppler ultrasound. *British Medical Bulletin*.

[26] MacPherson H., Altman D. G., Hammerschlag R. (2010). Revised STandards for Reporting Interventions in Clinical Trials of Acupuncture (STRICTA): extending the CONSORT statement. *PLOS Medicine*.

[27] Litscher G., Schikora D. (2002). Near-infrared spectroscopy for objectifying cerebral effects of needle and laserneedle acupuncture. *Spectroscopy*.

[28] Li H., Hou Z. W., Bai Y. L., Gu S. Z. (2011). Comparative study on curative effects of stroke treated with acupuncture by NIRS. *Zhongguo Zhen Jiu*.

[29] Li H., Hou Z.-W., Bai Y.-L., Gu S.-Z. (2010). Effects of electric-moxibustion on brain mantle accessed with near-infrared imaging. *Zhongguo Zhen Jiu*.

[30] Litscher G., Wang L. (2000). Cerebral near-infrared spectroscopy and acupuncture—preliminary results. *Biomedizinische Technik*.

[31] Litscher G., Wang L., Huber E. (2002). Changes in near-infrared spectroscopic parameters during manual stimulation with acupuncture needles. *Biomedizinische Technik*.

[32] Széles J. C., Litscher G. (2004). Objectivation of cerebral effects with a new continuous electrical auricular stimulation technique for pain management. *Neurological Research*.

[33] Litscher G., Schwarz G., Sandner-Kiesling A., Hadolt I., Eger E. (1998). Effects of acupuncture on the oxygenation of cerebral tissue. *Neurological Research*.

[34] Schwarz G., Litscher G., Sandner-Kiesling A. (2004). Pseudoparadoxical dissociation of cerebral oxygen saturation and cerebral blood flow velocity after acupuncture in a woman with cerebrovascular dementia: a case report. *Neurological Research*.

[35] Takamoto K., Hori E., Urakawa S. (2010). Cerebral hemodynamic responses induced by specific acupuncture sensations during needling at trigger points: a near-infrared spectroscopic study. *Brain Topography*.

[36] An Y., Jeon J. W., Kwon K., Choi C. (2014). Application of dynamic indocyanine green perfusion imaging for evaluation of vasoactive effect of acupuncture: a preliminary follow-up study on normal healthy volunteers. *Medical Devices: Evidence and Research*.

[37] Raith W., Pichler G., Sapetschnig I. (2013). Near-infrared spectroscopy for objectifying cerebral effects of laser acupuncture in term and preterm neonates. *Evidence-Based Complementary and Alternative Medicine*.

[38] Litscher G., Wang L., Schwarz G., Schikora D. (2005). Increases of intracranial pressure and changes of blood flow velocity due to acupressure, needle and laser needle acupuncture?. *Forsch Komplementärmed Klass Naturheilkd*.

[39] Litscher G., Ofner M., He W., Wang L., Gaischek I. (2013). Acupressure at the meridian acupoint Xiyangguan (GB33) influences near-infrared spectroscopic parameters (Regional oxygen saturation) in deeper tissue of the knee in healthy volunteers. *Evidence-Based Complementary and Alternative Medicine*.

[40] Ohkubo M., Hamaoka T., Niwayama M. (2009). Local increase in trapezius muscle oxygenation during and after acupuncture. *Dynamic Medicine*.

[41] Jimbo S., Atsuta Y., Kobayashi T., Matsuno T. (2008). Effects of dry needling at tender points for neck pain (Japanese: Katakori): near-infrared spectroscopy for monitoring muscular oxygenation of the trapezius. *Journal of Orthopaedic Science*.

[42] Banzer W., Hübscher M., Seib M., Vogt L. (2006). Short-time effects of laser needle stimulation on the peripheral microcirculation assessed by laser Doppler spectroscopy and near-infrared spectroscopy. *Photomedicine and Laser Surgery*.

[43] Hori E., Takamoto K., Urakawa S., Ono T., Nishijo H. (2010). Effects of acupuncture on the brain hemodynamics. *Autonomic Neuroscience: Basic and Clinical*.

[44] Litscher G. (2006). Bioengineering assessment of acupuncture, part 5: cerebral near-infrared spectroscopy. *Critical Reviews in Biomedical Engineering*.

[45] Chen G. S., Erdmann W. (1978). Effects of acupuncture on tissue oxygenation of the rat brain. *Southern Medical Journal*.

[46] Litscher G. (2002). Quantifizierung zerebraler Effekte der Ohrakupunktur durch innovative computergestützte Verfahren. *Der Akupunkturarzt/Aurikulotherapeut*.

[47] Huang W., Pach D., Napadow V. (2012). Characterizing acupuncture stimuli using brain imaging with fMRI—a systematic review and meta-analysis of the literature. *PLoS ONE*.

